# Research trends on lymphedema after mastectomy for breast cancer patients from 2000 to 2023: a scientometric analysis

**DOI:** 10.3389/fonc.2025.1440966

**Published:** 2025-02-04

**Authors:** Ling Chen, Yuxian Zheng, Daitian Zheng, Zhiyang Li, Hongwu Chen, Chujun Chen, Shuxian Yu

**Affiliations:** ^1^ Breast Center, Cancer Hospital of Shantou University Medical College, Guangdong, Shantou, China; ^2^ Nursing Department, Shantou University Medical College, Guangdong, Shantou, China; ^3^ Department of General Surgery, The Second Affiliated Hospital of Shantou University Medical College, Shantou, China; ^4^ Department of Neurosurgery, The First Affiliated Hospital of Shantou University Medical College, Shantou, China; ^5^ Nursing Department, Cancer Hospital of Shantou University Medical College, Guangdong, Shantou, China

**Keywords:** bibliometric analysis, breast cancer-related lymphedema, arm lymphedema, self-management, rehabilitation, quality of life, prevention

## Abstract

**Background:**

Breast cancer-related lymphedema (BCRL) is a common and debilitating complication following breast cancer treatment. Despite its significant impact on patients’ quality of life, bibliometric analyses focusing on BCRL are scarce. This study aims to explore global research trends on BCRL from 2000 to 2023, identify existing knowledge gaps, and highlight emerging focus areas through a bibliometric approach.

**Methods:**

A comprehensive search was conducted using the Web of Science (WOS) database to retrieve literature published between January 2000 and November 2023. Bibliometric analyses and visualizations were performed using R Studio, CiteSpace, and VOSviewer. Key data extracted included publication trends, contributing countries and institutions, leading authors, journals, research categories, and keywords. Outcome measures for analysis included the number of publications, citation counts, author productivity, and keyword co-occurrence.

**Results:**

A total of 919 eligible publications from 52 countries and regions, 1,163 institutions, and 3,550 authors were identified. These publications appeared in 255 journals, with “*Lymphology*” emerging as the journal with the highest citation count. The USA was the most prolific contributor to the field. The annual number of publications demonstrated a consistent upward trend. Keyword co-occurrence analysis revealed prominent research hotspots, including “lymphedema,” “women,” “breast cancer,” “arm lymphedema,” and “quality of life.” Emerging keyword trends from 2021 to 2023 highlighted “prevention” and “validity” as pivotal research frontiers.

**Conclusions:**

This bibliometric study highlights the growing interest in breast cancer-related lymphedema research and identifies key areas for future investigation, including prevention, diagnosis, and treatment strategies. The results underscore the need for further exploration of these emerging research areas to improve patient outcomes.

## 1 Introduction

Lymphedema, defined as tissue swelling caused by the abnormal accumulation of protein-rich fluid, results from lymphatic system dysfunction (1). It is a common secondary complication of cancer treatments, particularly following breast cancer therapies (2). Approximately one in five breast cancer survivors develop arm lymphedema (3), with a cumulative incidence of 11.9% observed in a study of 5,549 patients within five years post-surgery (4). In the United States, the incidence of breast cancer peaks among white women around age 80, whereas in Asian countries, rates tend to plateau or decline after age 50 (5). This condition is often triggered by lymphatic damage caused by surgical interventions such as mastectomy and axillary lymph node dissection or by radiotherapy (6, 7). Additional risk factors, such as elevated body mass index and advanced cancer stages (8), further contribute to the development of breast cancer-related lymphedema (BCRL) (9).

BCRL can affect the affected side’s arm, hand, finger, breast, or body. With the arm being the most commonly impacted area. Symptoms include pain, swelling, and restricted joint mobility (10), and complications may include recurrent infections, skin fibrosis, and chronic pain. These symptoms often progress and may become irreversible (11). BCRL significantly impairs functioning, work performance, and social interactions (12), imposing a substantial economic burden (12). Additionally, the condition adversely affects mental health, leading to emotional distress, reduced self-esteem, social isolation, and dissatisfaction with physical appearance (13). For individuals in physically demanding occupations, BCRL reduces work capacity and further diminishes their quality of life (14, 15). Effective management involves early symptom detection, clinical assessment, and objective measurements (16). Preventive strategies, including risk assessment, patient education, and early intervention, are essential for mitigating symptoms and improving quality of life (17–19).

As the most commonly diagnosed cancer worldwide, breast cancer poses a significant public health challenge (20), with BCRL being a critical concern (21). Despite advances in understanding and managing BCRL, research gaps persist, particularly regarding the integration of clinical practices with global research trends. Addressing these gaps is crucial for developing comprehensive clinical guidelines and improving patient care. Bibliometric analysis, which quantitatively evaluates academic literature, provides valuable insights into research productivity, thematic priorities, and collaborative networks within a field (22). This study employs bibliometric methods to examine the evolution of BCRL research, identify emerging themes, and analyze collaboration patterns (23). The findings aim to guide researchers, clinicians, and policymakers by highlighting areas requiring further investigation, ultimately contributing to better outcomes for breast cancer survivors.

## 2 Methods

### 2.1 Data source and processing

On November 30, 2023, data on the correlation between lymphedema and breast cancer were extracted from the Web of Science (WOS) database, which provides access to billions of cited references across various disciplines (24). The search covered publications from January 1, 2000, to November 30, 2023. The search parameters were configured as follows: Search In = “Web of Science Core Collection” and Editions = “Science Citation Index Expanded (SCI-EXPANDED)–1975-present.” Medical Subject Headings (MeSH) terms and entry terms such as “exosome” and “breast cancer” were employed as part of the search strategy. The search query was structured as follows: TS=(“Breast Neoplasm” OR “Breast Tumor” OR “Breast Cancer” OR “Mammary Cancer” OR “Breast Malignant Neoplasm” OR “Breast Malignant Tumor” OR “Breast Carcinoma”) AND TS=(“Breast Cancer Lymphedema” OR “Breast Cancer Treatment Related Lymphedema” OR “Breast Cancer Related Arm Lymphedema” OR “Breast Cancer Related Lymphedema” OR “Postmastectomy Lymphedema” OR “Post-mastectomy Lymphedema”). This search yielded 1,103 records. To refine the dataset, document types were limited to articles and reviews, and the language was restricted to English. After a detailed review, the dataset was reduced to 919 records, comprising 758 articles and 161 review articles. Exclusions were made based on specific criteria, including 102 meeting abstracts, 35 editorial materials, 26 letters, nine corrections, one book review, one expression of concern, and ten non-English publications. No retracted articles were identified using Zotero software. Ultimately, the final dataset consisted of 919 publications, with 758 articles (82.48%) and 161 review articles (17.52%).

### 2.2 Data analysis

**Figure 1:**
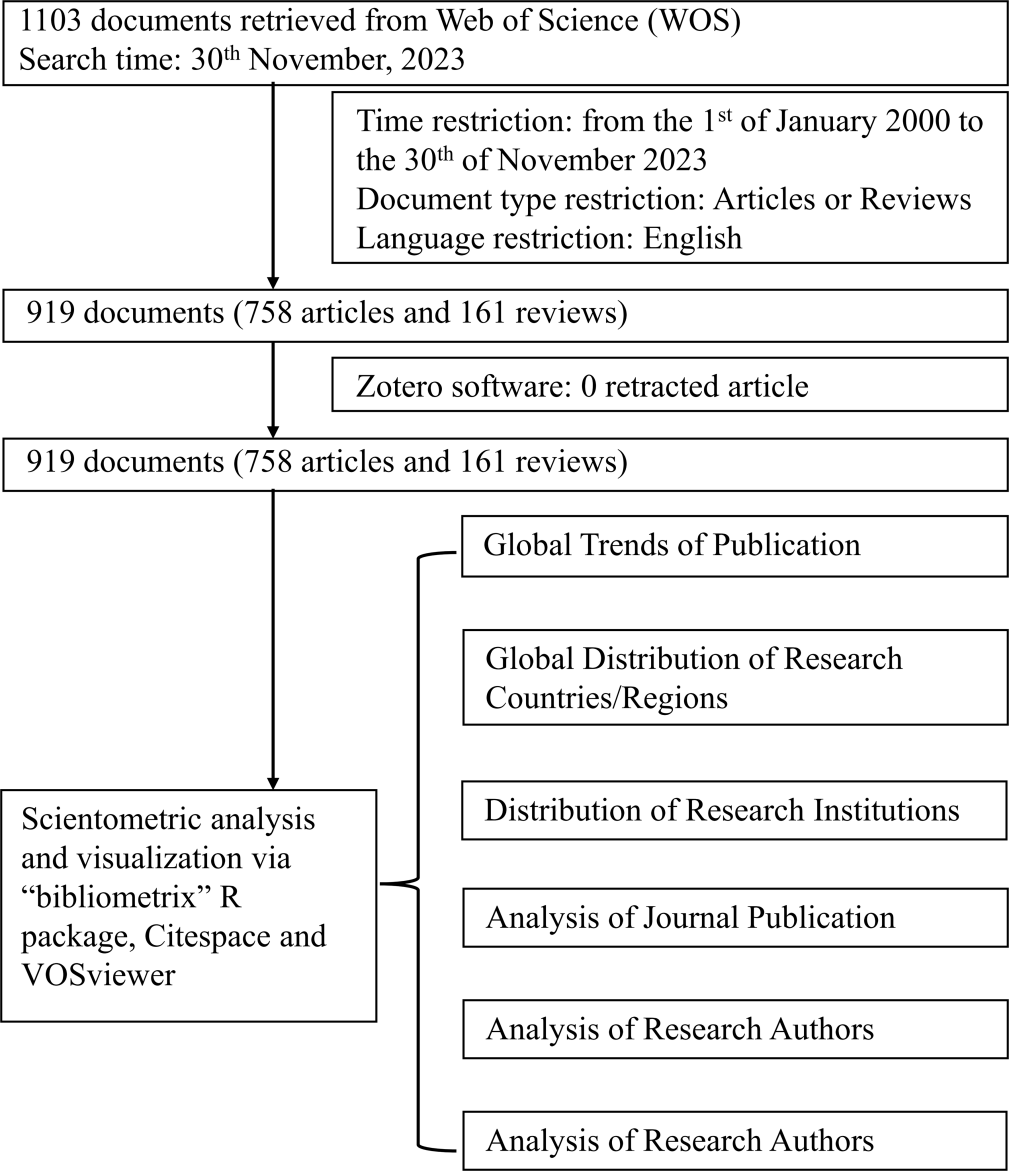
Flowchart of the records collection and analysis process.

The “Analyze Results” function in WOS was utilized to extract preliminary information, including publication years, document types, research areas, authors, affiliations, journals, publishers, countries/regions, languages, funding agencies, and open access status. For deeper analysis, the R package bibliometrix (version 4.3.1) was used to compute annual publication counts, analyze trending topics, and generate collaborative and thematic maps (25). CiteSpace (version 6.1), a visualization software developed by Chaomei Chen (27), was employed to conduct visual analyses, including identifying the top 50 references with the strongest citation bursts and performing dual-map overlay analyses. VOSviewer (version 1.6.18) was used to construct co-citation maps, analyze co-authorship patterns, and evaluate keyword co-occurrence networks. It also assessed collaboration networks among countries, institutions, and authors (26). Nodes in the visualizations represent individual entities, with colors indicating cluster membership. The size of each node reflects the count or frequency of entities within the cluster while connecting lines illustrate the strength of collaboration or co-occurrence between entities (27). The complete retrieval strategy, the number of identified records, and the analysis process are illustrated in **Figure 1**.

## 3 Results

### 3.1 Global publication trends

**Figure 2:**
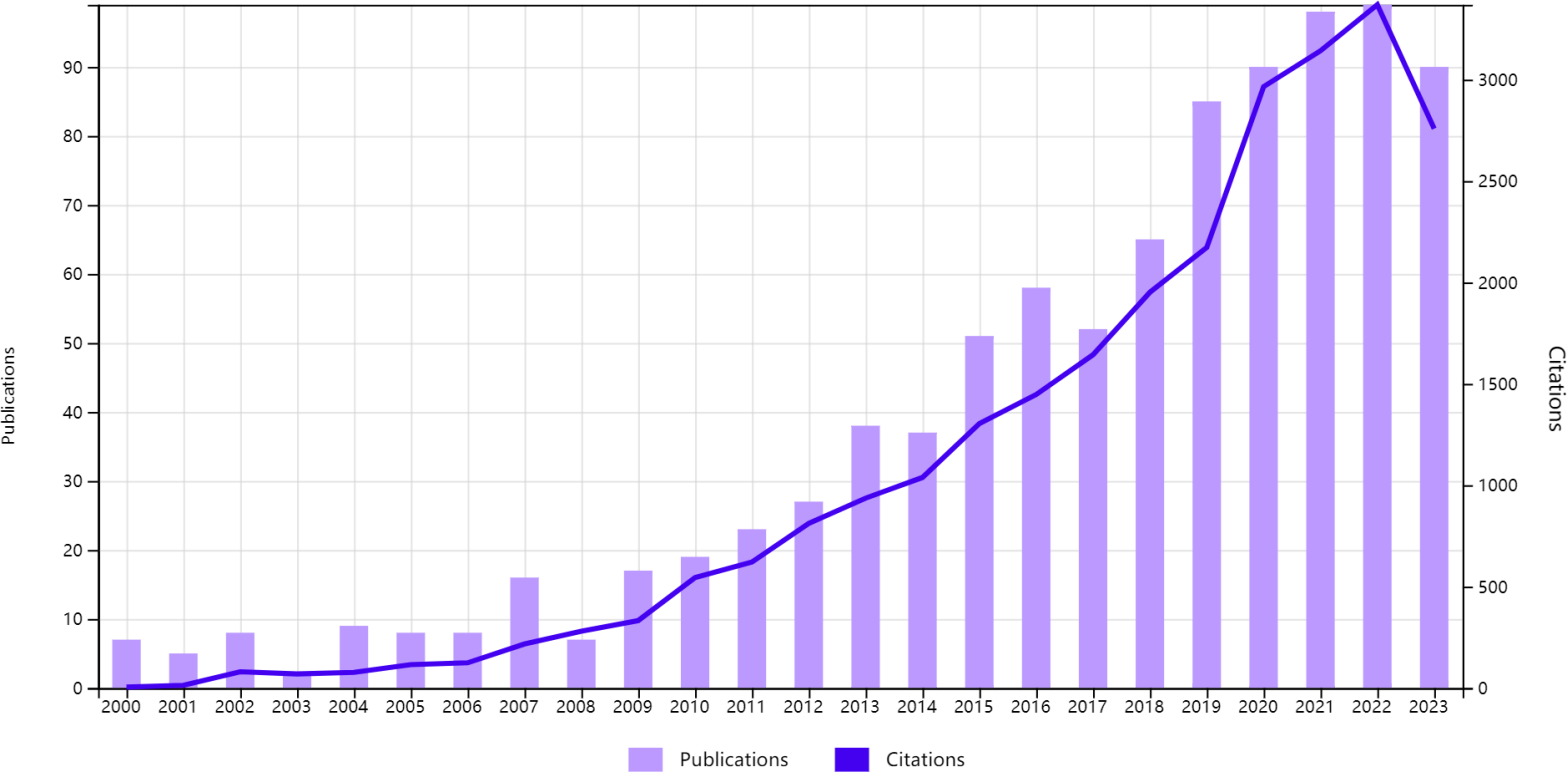
Trends in the number of publications and citations.

From January 1, 2000, to November 30, 2023, a total of 919 publications related to BCRL were retrieved from the WOS. This dataset included 758 articles (82.48%) and 161 review articles (17.52%). The annual distribution of these publications is presented in **Table 1**. As shown in **Figure 2**, the number of publications steadily increased from 7 (0.76%) in 2000 to a peak of 99 (10.77%) in 2022. The apparent decrease in 2023 reflects the data collection cutoff date of November 30, 2023. On average, 40 papers were published annually, with an average growth rate of 12.81%. Both annual and cumulative publication trends demonstrate exponential growth, underscoring the rapid development and heightened interest in this research area.

**Table 1:** The number of publications each year.

Publication years	Record count	% of 919
2000	7	0.762
2001	5	0.544
2002	8	0.871
2003	2	0.218
2004	9	0.979
2005	8	0.871
2006	8	0.871
2007	16	1.741
2008	7	0.762
2009	17	1.85
2010	19	2.067
2011	23	2.503
2012	27	2.938
2013	38	4.135
2014	37	4.026
2015	51	5.55
2016	58	6.311
2017	52	5.658
2018	65	7.073
2019	85	9.249
2020	90	9.793
2021	98	10.664
2022	99	10.773
2023	90	9.793

### 3.2 Global distribution of research countries/regions

**Figure 3:**
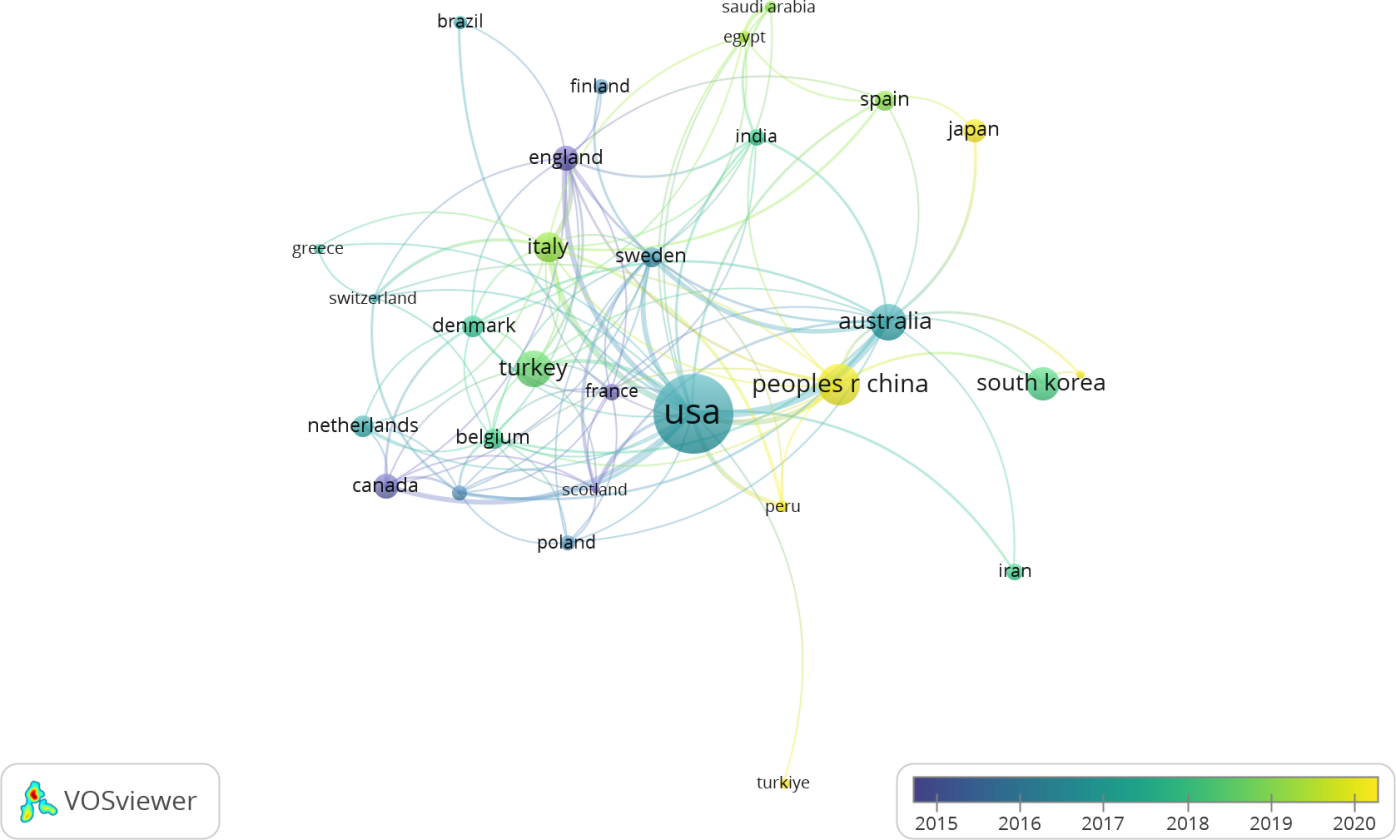
The collaborative network between countries/regions.

**Figure 4:**
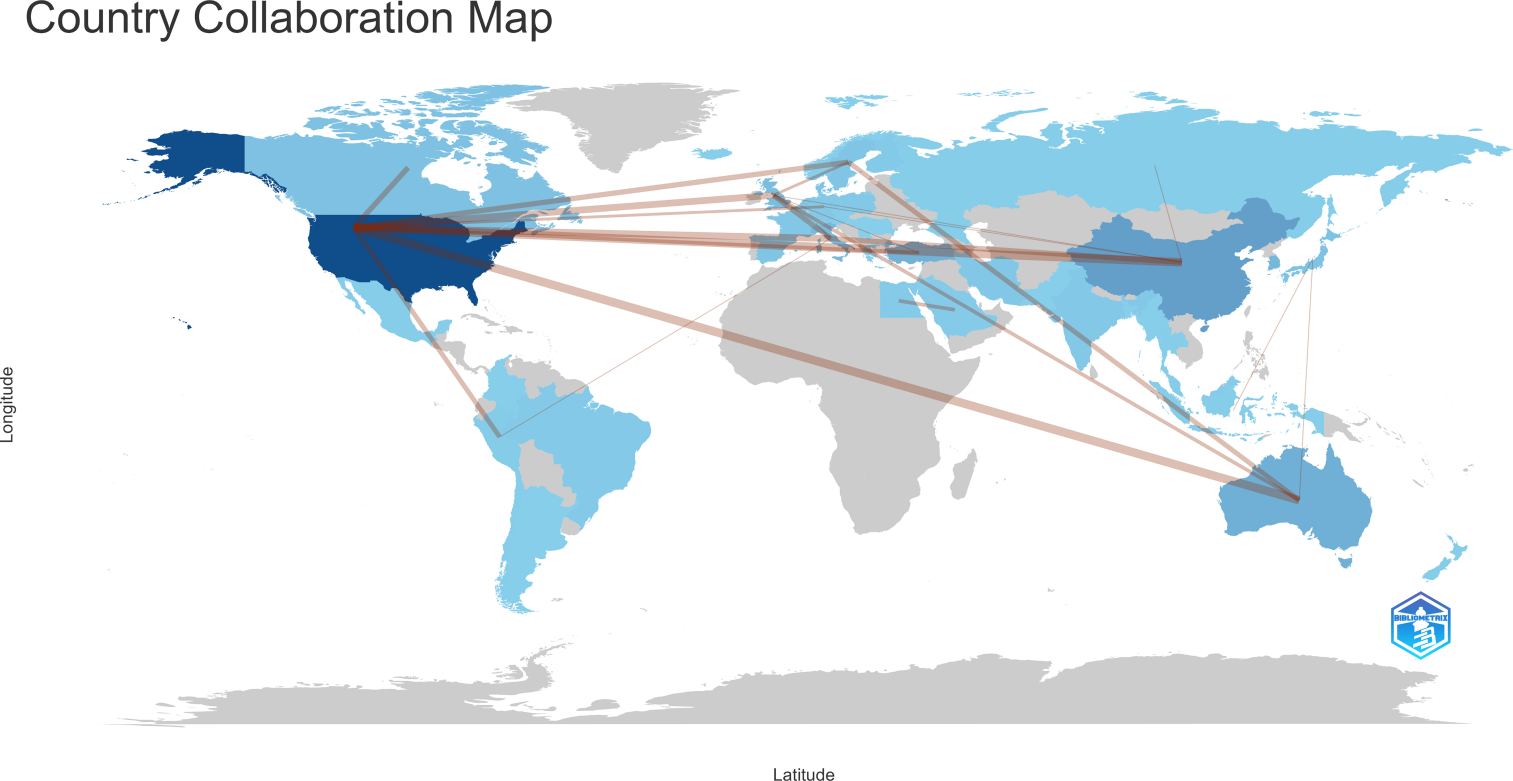
The collaboration world map.

Between January 1, 2000, and November 30, 2023, 52 countries/regions contributed to this field. The top 25 contributors are detailed in **Table 2**, with the USA leading with 341 publications (37.11%), followed by China (90 publications, 9.79%) and Australia (75 publications, 8.16%). Among 29 countries/regions with at least five publications, an international collaborative network was identified (**Figure 3**). This network highlights extensive global collaboration, particularly among the USA, UK, Italy, and Australia. Initially, research was concentrated in developed countries such as England and Canada but later expanded to include the USA and Asian nations, especially China and Japan. **Figure 4** presents a collaborative world map, which visually represents publication volumes and collaborative ties, with the USA dominating this research domain.

**Table 2:** The top 25 countries or regions with the most publications.

Countries/regions	Record count	Percentage	Total citation	Average article citations
USA	341	37.106	12351	42.00
China (Mainlands)	90	9.793	2060	18.20
Australia	75	8.161	1812	35.50
Turkey	71	7.726	746	11.10
South Korea	58	6.311	665	11.90
Italy	50	5.441	709	21.50
England	38	4.135	1090	43.60
Canada	32	3.482	919	38.30
Taiwan	31	3.373	1080	21.00
Japan	29	3.156	319	11.80
Denmark	26	2.829	507	22.00
Netherlands	25	2.720	698	31.70
Belgium	24	2.612	322	16.10
Spain	24	2.612	235	12.40
Sweden	23	2.503	281	21.60
France	17	1.850	665	51.20
Iran	17	1.850	291	17.10
India	15	1.632	85	10.60
Germany	14	1.523	256	32.00
Poland	14	1.523	102	7.80
Finland	13	1.415	680	56.70
Brazil	10	1.088	205	20.50
Egypt	9	0.979	100	16.70
Saudi Arabia	8	0.871	70	14.00
Peru	7	0.762	16	5.30

### 3.3 Distribution of research institutions

**Figure 5:**
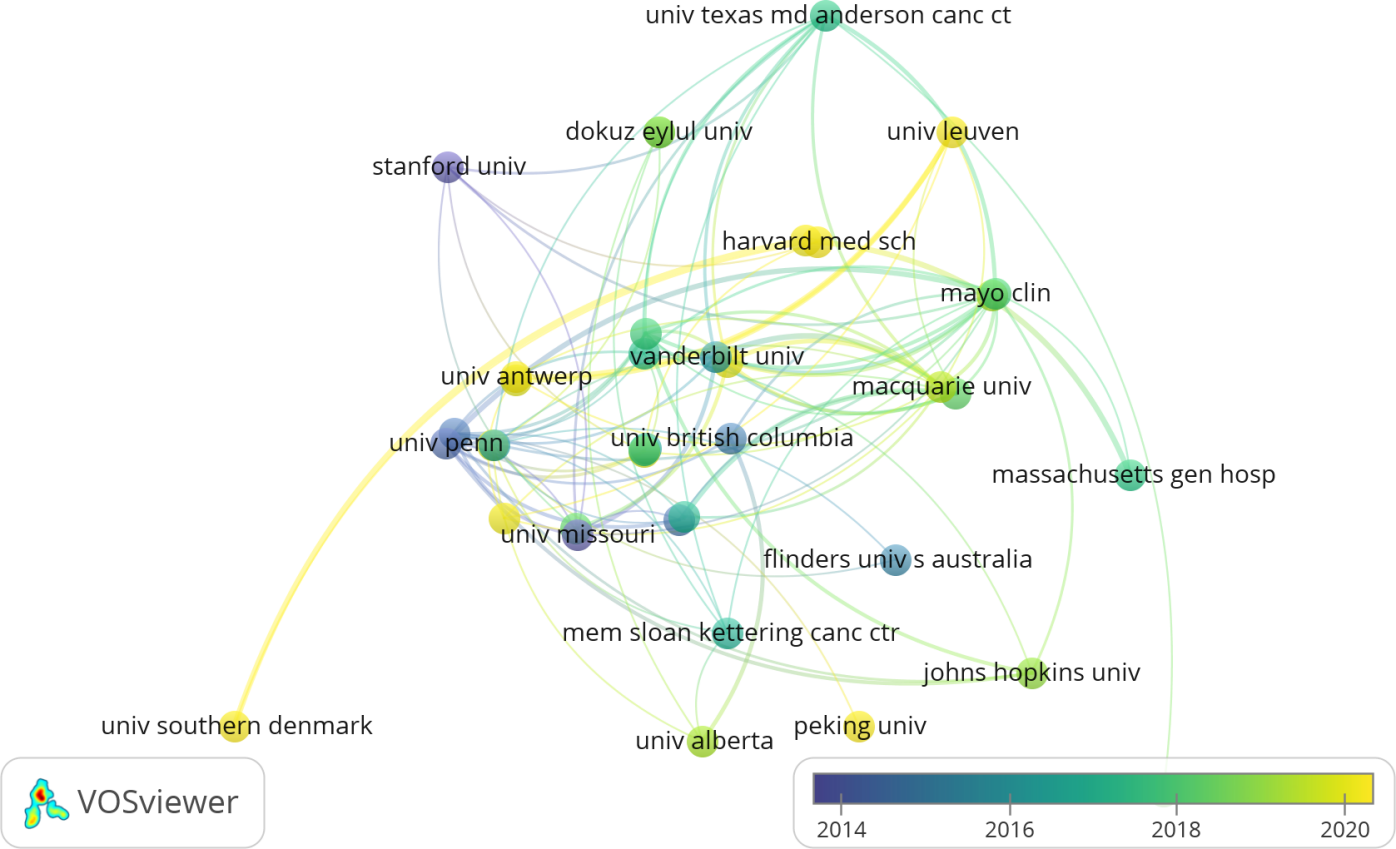
The collaborative network between institutions.

Between January 1, 2000, and November 30, 2023, 1,163 institutions were active in BCRL research. Thirteen institutions published 20 or more articles, with Harvard University (50 publications, 5.44%) and the University of Texas System (40 publications, 4.35%) leading the field (**Table 3**). Collaborative networks among 46 institutions with at least eight publications each are illustrated in **Figure 5**. Larger circles in the network represent institutions with higher publication counts, while the number of connecting lines reflects the degree of collaboration. Leading institutions included Vanderbilt University (34 publications), UT MD Anderson Cancer Center (32 publications), Mayo Clinic (29 publications), University of Pennsylvania (26 publications), and Memorial Sloan Kettering Cancer Center (23 publications). Recently, institutions such as Stanford University, the University of Pennsylvania, and the University of Missouri have initiated new studies, while the University of Southern Denmark and Peking University have emerged as active contributors.

**Table 3:** The 25 institutions with the most publications.

Affiliations	Record count	Percentage
Harvard University	50	5.441
University of Texas System	40	4.353
Ut Md Anderson Cancer Center	34	3.700
Vanderbilt University	34	3.700
Mayo Clinic	31	3.373
Harvard Medical School	28	3.047
Massachusetts General Hospital	28	3.047
University of Pennsylvania	27	2.938
University of Sydney	24	2.612
Memorial Sloan Kettering Cancer Center	23	2.503
Macquarie University	22	2.394
Cleveland Clinic Foundation	21	2.285
Seoul National University SNU	20	2.176
Lund University	19	2.067
Pennsylvania Commonwealth System of Higher Education PCSHE	19	2.067
University of Missouri Columbia	19	2.067
University of Missouri System	19	2.067
Pennsylvania Medicine	18	1.959
University of California System	18	1.959
Ku Leuven	17	1.850
Skane University Hospital	17	1.850
Hacettepe University	16	1.741
New York University	15	1.632
University of Queensland	15	1.632
University of Southern Denmark	14	1.523

### 3.4 Analysis of journals

**Figure 6:**
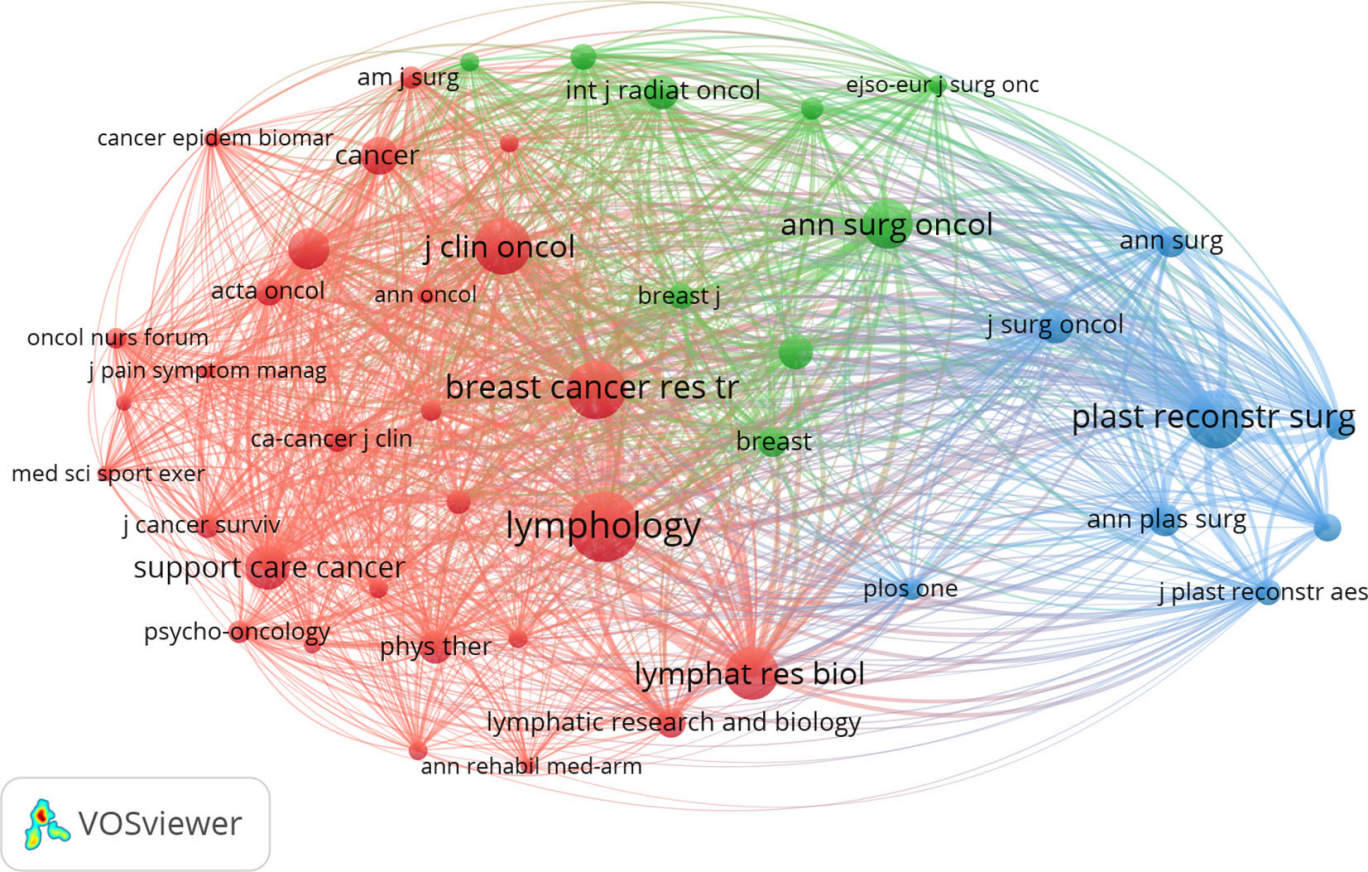
The network of journals with cocitation.

A total of 255 journals published research on BCRL. **Table 4** lists the top ten journals by publication count, led by *Lymphatic Research and Biology* with 113 publications, followed by *Supportive Care in Cancer* (n=48) and *Breast Cancer Research and Treatment* (n=44). Journals such as *Annals of Surgical Oncology*, *Plastic and Reconstructive Surgery*, and *Annals of Plastic Surgery* primarily focus on surgical aspects of BCRL. *Breast Cancer Research and Treatment* had the highest citation count (1,544 citations), followed by *Supportive Care in Cancer* (1,420 citations). Cocitation analysis, conducted with a citation threshold of 120, identified 45 sources, with *Lymphology* as the most cited journal, followed by *Breast Cancer Research and Treatment* and *Plastic and Reconstructive Surgery* (**Table 5**). A network map (**Figure 6**) revealed three clusters, with *Breast Cancer Research and Treatment* forming the central node of the red cluster, which had the highest total link strength.

**Table 4:** The citations of the top 10 most popular journals.

Sources	Documents	Citations	Total link strength
Lymphatic Research and Biology	113	1351	640
Supportive Care in Cancer	48	1420	435
Breast Cancer Research and Treatment	44	1544	590
Lymphology	40	1062	298
Annals of Surgical Oncology	26	1015	399
Plastic and Reconstructive Surgery	23	867	260
Annals of Plastic Surgery	21	746	201
Cancer	18	1220	240
Journal of Cancer Survivorship	16	719	188
Journal of Reconstructive Microsurgery	14	247	144

**Table 5:** The cocitation of the top 10 most popular journals.

Source	Citations	Total link strength
Lymphology	1827	41701
Breast Cancer Research and Treatment	1287	37420
Plastic and Reconstructive Surgery	1267	32294
Journal of Clinical Oncology	1181	34843
Lymphatic Research and Biology	1073	24865
Annals of Surgical Oncology	978	31557
Supportive Care in Cancer	796	19108
CANCER-American Cancer Society	700	19568
Cancer	598	14404
International Journal of Radiation Oncology Biology Physics	501	14887

### 3.5 Analysis of research authors

**Figure 7:**
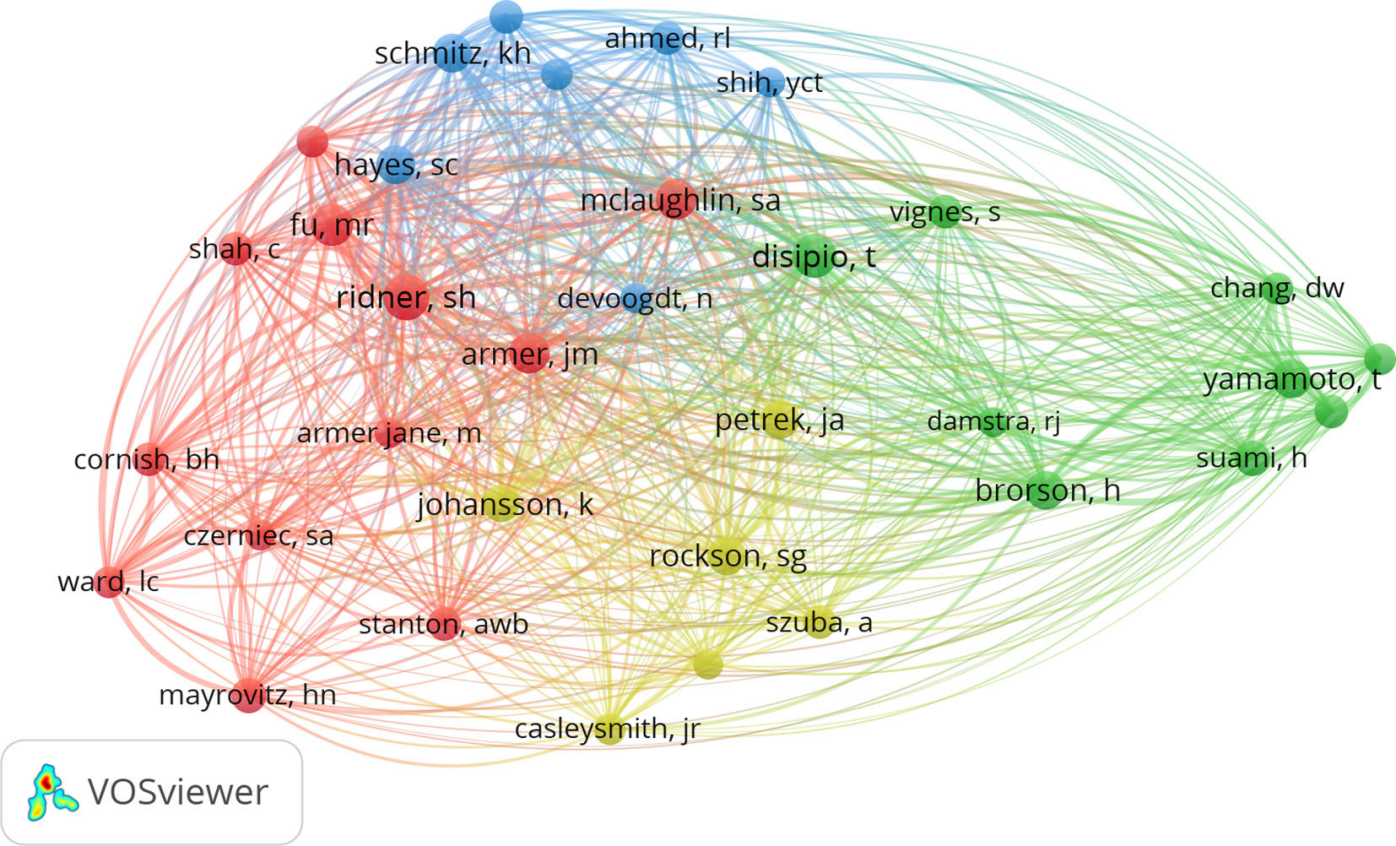
The network of authors with cocitation.

Between January 1, 2000, and November 30, 2023, 3,550 authors contributed to BCRL research (**Table 6**). The most prolific authors were Taghian, Alphonse G. (24 publications), Schmitz, Kathryn H., and Ridner, Sheila (22 publications each). Scientific productivity and impact were assessed using the h-index, g-index, and m-index (28). Taghian, Alphonse G. achieved the highest h-index with 16, g-index, and m-index among all authors. A cocitation network map (**Figure 7**) revealed collaborative patterns among these researchers.

**Table 6:** The top 25 authors with the most publications.

Authors	Record count	Percentage	H index	G index	M index	Total citation
Taghian, Alphonse G.	24	2.612	16	24	1.231	918
Schmitz, Kathryn H.	22	2.394	16	22	1.000	1826
Ridner, Sheila	22	2.394	14	22	0.737	1212
Brunelle, Cheryl L.	18	1.959	10	18	1.111	538
Shah, Chirag	17	1.85	13	17	1.000	630
Armer, Jane M.	16	1.741	14	16	0.667	1299
Fu, Mei R.	15	1.632	11	15	0.579	399
Boyages, John	15	1.632	10	15	1.111	408
Devoogdt, Nele	15	1.632	8	14	0.571	199
Kilbreath, Sharon L.	13	1.415	9	13	0.643	496
Sørensen, Jens Ahm	13	1.415	7	12	0.778	154
De Vrieze, Tessa	12	1.306	6	10	1.000	106
Dietrich, Mary S.	12	1.306	10	12	0.625	455
Singhal, Dhruv	12	1.306	–	–	–	–
Skolny, Melissa N.	12	1.306	11	12	0.917	669
Suami, Hiroo	12	1.306	11	12	0.647	544
Vicini, Frank	12	1.306	10	12	0.833	453
Ciudad, Pedro	11	1.197	7	11	0.778	231
Gebruers, Nick	11	1.197	7	11	0.875	170
Invernizzi, Marco	11	1.197	9	11	1.800	243
Miller CL	10	1.088	9	10	0.75	568
Gillespie, Tessa C.	10	1.088	6	10	1.000	238
Koelmeyer, L.	10	1.088	6	10	0.875	253
Mehrara, Babak Joseph	10	1.088	–	–	–	–
De Sire A	10	1.088	–	–	–	–

### 3.6 Analysis of publications

**Figure 8:**
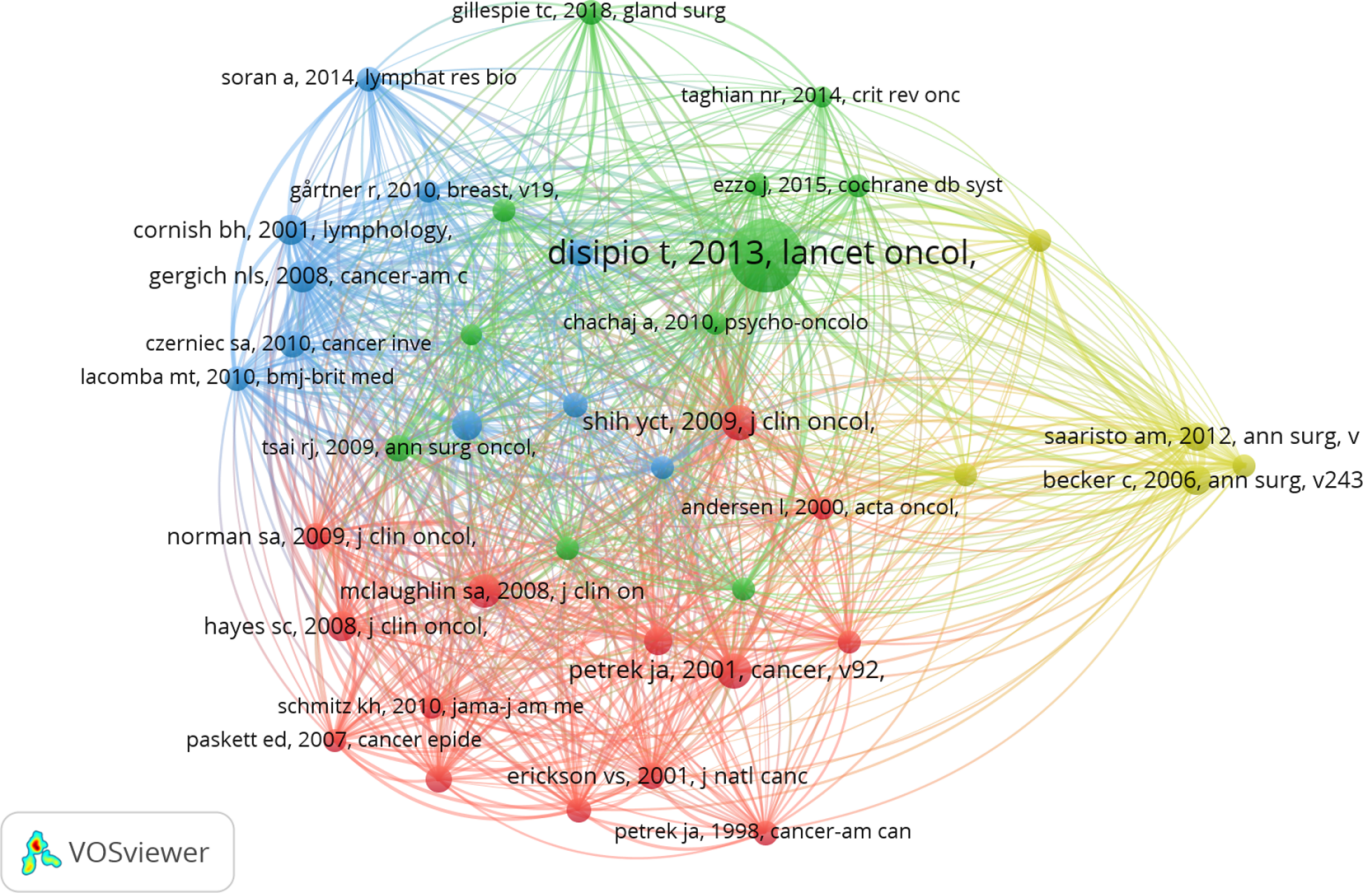
The network of references with cocitation.

**Figure 9:**
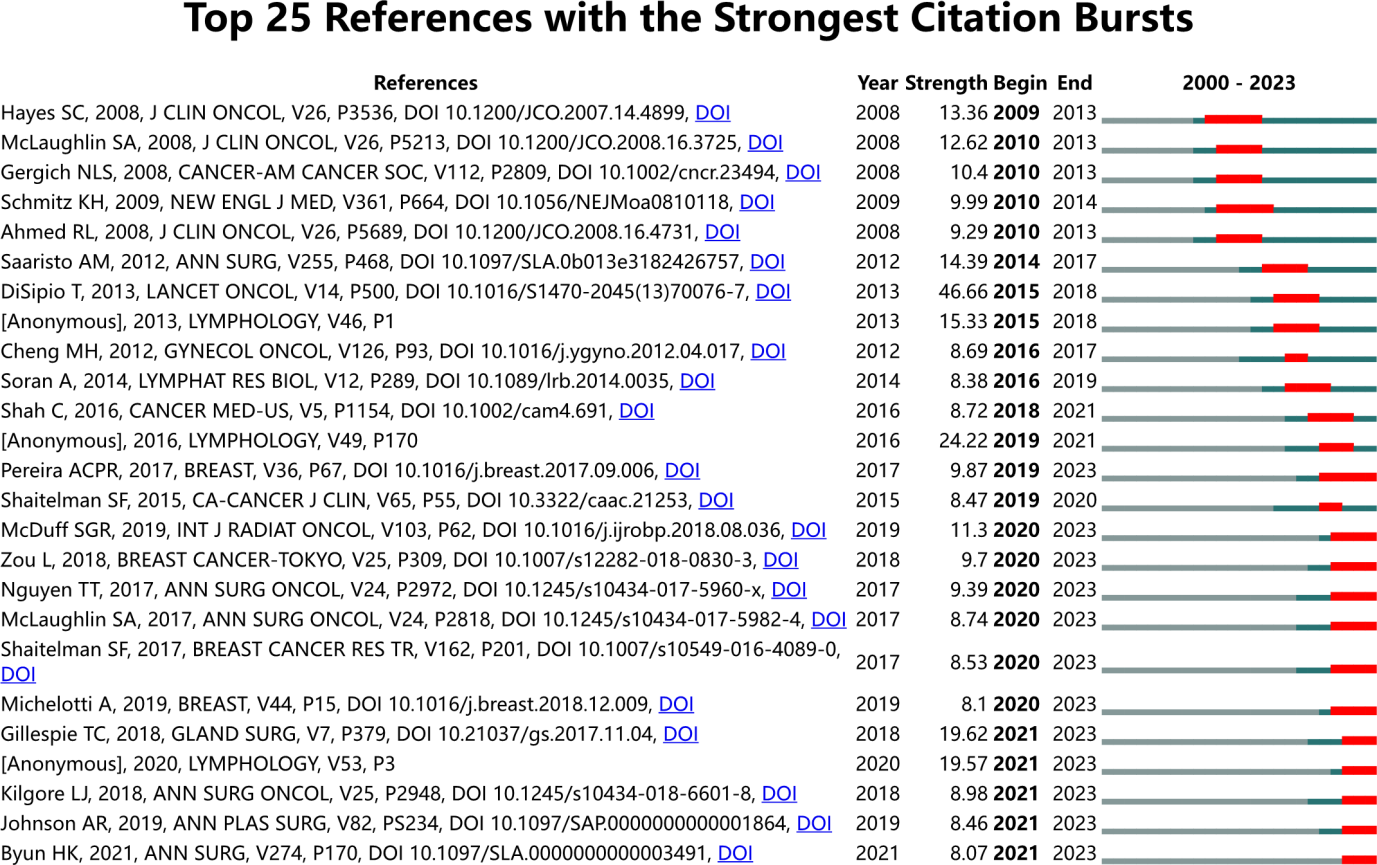
Top 25 references with the strongest citation bursts.

The top 10 publications ranked by global citation count are listed in **Table 7**. Anne G. Warren’s “Lymphedema: A Comprehensive Review,” published in *Annals of Plastic Surgery* in 2007, received the highest citation count, providing a systematic approach to evaluating and managing lymphedema patients. Schmitz, Kathryn H. authored two randomized controlled trials on the safety of weightlifting in women with BCRL. Cocitation analysis using VOSviewer identified 93,571 references across four clusters (**Figure 8**). The most frequently cited reference was Tracey DiSipio’s “Incidence of unilateral arm lymphoedema after breast cancer: a systematic review and meta-analysis,” published in *Lancet Oncology* in 2013. This study, which included 72 articles, reported an overall arm lymphedema incidence of 17%. Citation burst analysis (**Figure 9**) identified references such as “Risk of Lymphedema Following Contemporary Treatment for Breast Cancer” as having ongoing influence in the field.

**Table 7:** The top 10 most citations about BCRL studies.

Documents	Author	Journal	DOI	Year	Global citations
Lymphedema: A Comprehensive Review (9)	Anne G. Warren	Annals of Plastic Surgery	10.1097/01.sap.0000257149.42922.7e	2007	447
Arm edema in breast cancer patients (45)	Virginia S. Erickson	JNCI-Journal of the National Cancer Institute	10.1093/jnci/93.2.96	2001	383
Weight lifting in women with breast-cancer-related lymphedema (32)	Schmitz, Kathryn H.	The New England Journal of Medicine	10.1056/NEJMoa0810118	2009	381
Postmastectomy lymphedema: long-term results following microsurgical lymph node transplantation (46)	Becker Corinne	Annals of Surgery	10.1097/01.sla.0000201258.10304.16	2006	354
Incidence, treatment costs, and complications of lymphedema after breast cancer among women of working age: a 2-year follow-up study (47)	Ya-Chen Tina Shih	Journal of Clinical Oncology	10.1200/JCO.2008.18.3517	2009	340
Lymphedema and quality of life in breast cancer survivors: the Iowa Women’s Health Study (48)	Rehana L Ahmed	Journal of Clinical Oncology	10.1200/JCO.2008.16.4731	2008	274
Weight lifting for women at risk for breast cancer-related lymphedema: a randomized trial (31)	Schmitz, Kathryn H.	JAMA- The Journal of the American Medical Association	10.1001/jama.2010.1837	2010	274
Lymphedema: a primer on the identification and management of a chronic condition in oncologic treatment (2)	Brian D Lawenda	CA-A Cancer Journal for Clinicians	10.3322/caac.20001	2009	257
Upper-body morbidity after breast cancer: incidence and evidence for evaluation, prevention, and management within a prospective surveillance model of care (49)	Sandra C Hayes	CANCER-American Cancer Society	10.1002/cncr.27467	2012	249
Microvascular breast reconstruction and lymph node transfer for postmastectomy lymphedema patients (50)	Anne M Saaristo	Annals of Surgery	10.1097/SLA.0b013e3182426757	2012	231

### 3.7 Analysis of keywords

**Figure 10:**
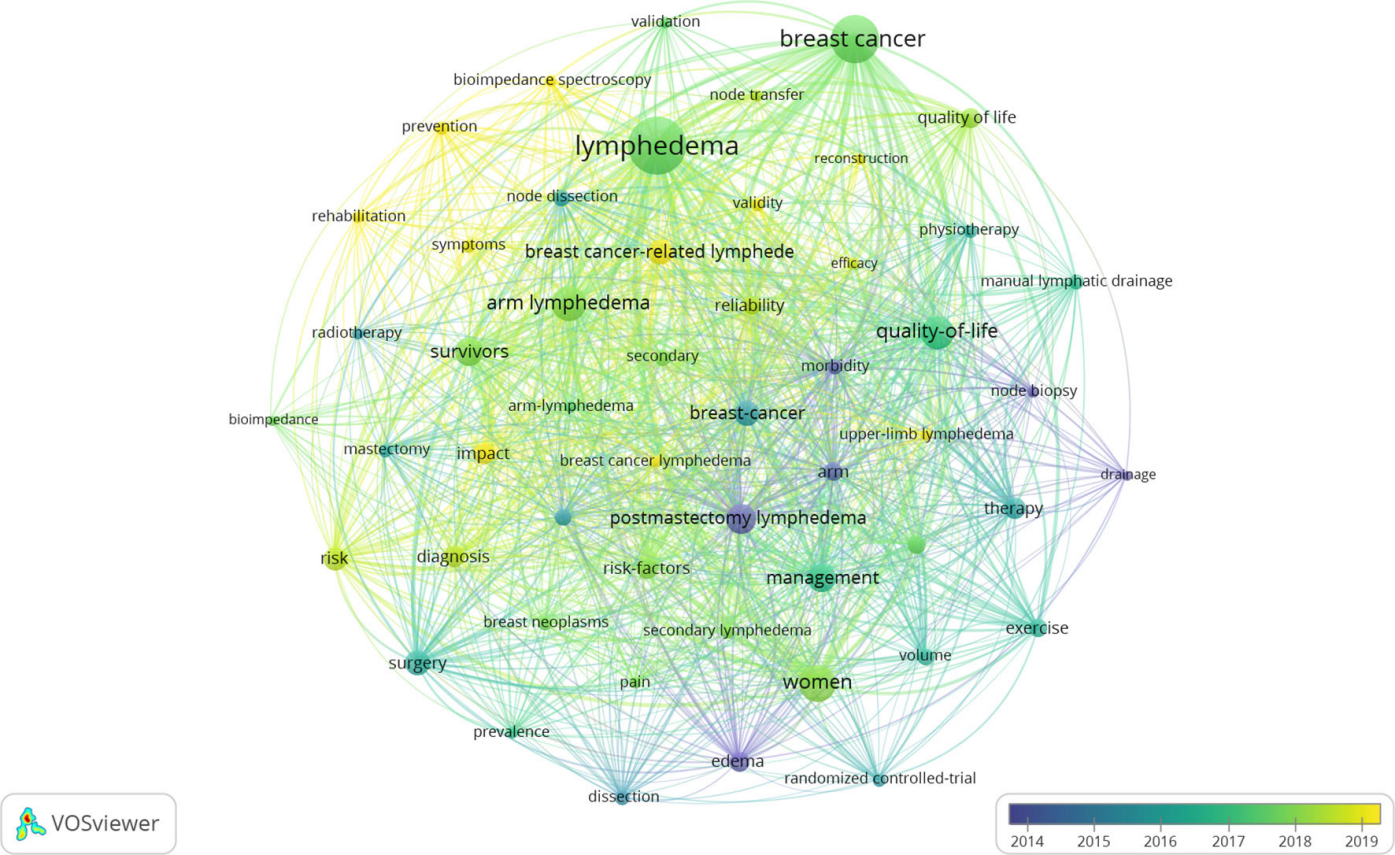
The network of cooccurring keywords.

A total of 2,184 keywords were extracted from the collected records, providing insights into thematic evolution and research hotspots in breast cancer-related lymphedema (BCRL). The top 25 most frequently used keywords are presented in **Table 8**. Using VOSviewer, the 52 most common keywords were visualized with a minimum occurrence threshold set at 30 (**Figure 10**). Deeper shades in the visualization represent more frequently occurring keywords, while proximity to the central yellow block indicates higher citation frequency and relevance. Frequently appearing keywords included “lymphedema,” “breast cancer,” and “women,” underscoring their significance in the field over the past two decades. Other prominent terms included “arm lymphedema,” “quality of life,” and “postmastectomy lymphedema”. Co-occurrence analysis identified five thematic clusters: management and quality of life (15 in red), women’s surgery (13 in green), symptoms and validity (11 in blue), therapy (7 in yellow), and breast cancer-related lymphedema (6 in purple).

**Table 8:** Top 25 most frequent words.

Words	Occurrences
women	222
arm lymphedema	199
quality-of-life	181
postmastectomy lymphedema	155
survivors	142
management	135
breast-cancer	117
risk	105
surgery	103
risk-factors	96
therapy	90
impact	89
diagnosis	78
edema	74
arm	70
cancer-related lymphedema	68
reliability	68
axillary dissection	63
node dissection	58
secondary	58
volume	53
morbidity	52
exercise	49
dissection	48
secondary lymphedema	39

**Figure 11:**
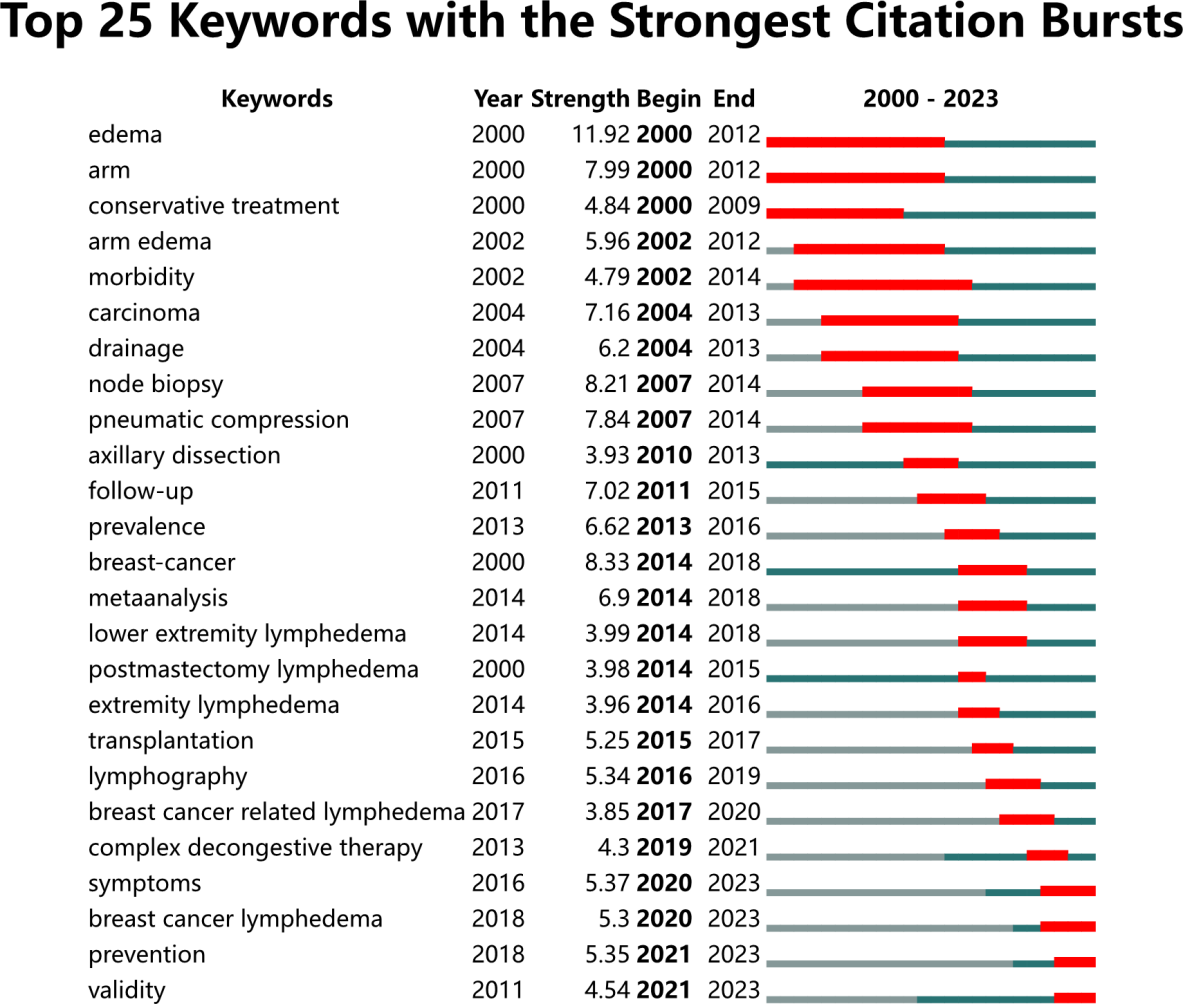
Top 25 keywords with the strongest citation bursts.

To explore emerging terms and research trends, an analysis of burst term keywords characterized by delayed emergence and extended influence was conducted. Twenty-five keywords with the strongest bursts were identified (**Figure 11**). The figure includes authors, publication years, and catalog information on the left, while burst-related metrics (burst value, start year of attention, and decline year) are listed on the right. From 2000 to 2023, significant keywords included “edema” (2000–2012), “arm” (2000–2012), “conservative treatment” (2000–2009), “arm edema” (2002–2012), “morbidity” (2002–2014), “carcinoma” (2004–2013), “drainage” (2004–2013), and “node biopsy” (2007–2014). Over the past 23 years, terms such as “edema,” “breast cancer,” and “node biopsy” displayed the most intense bursts, highlighting their prominence and marking pivotal areas for future research.

**Figure 12:**
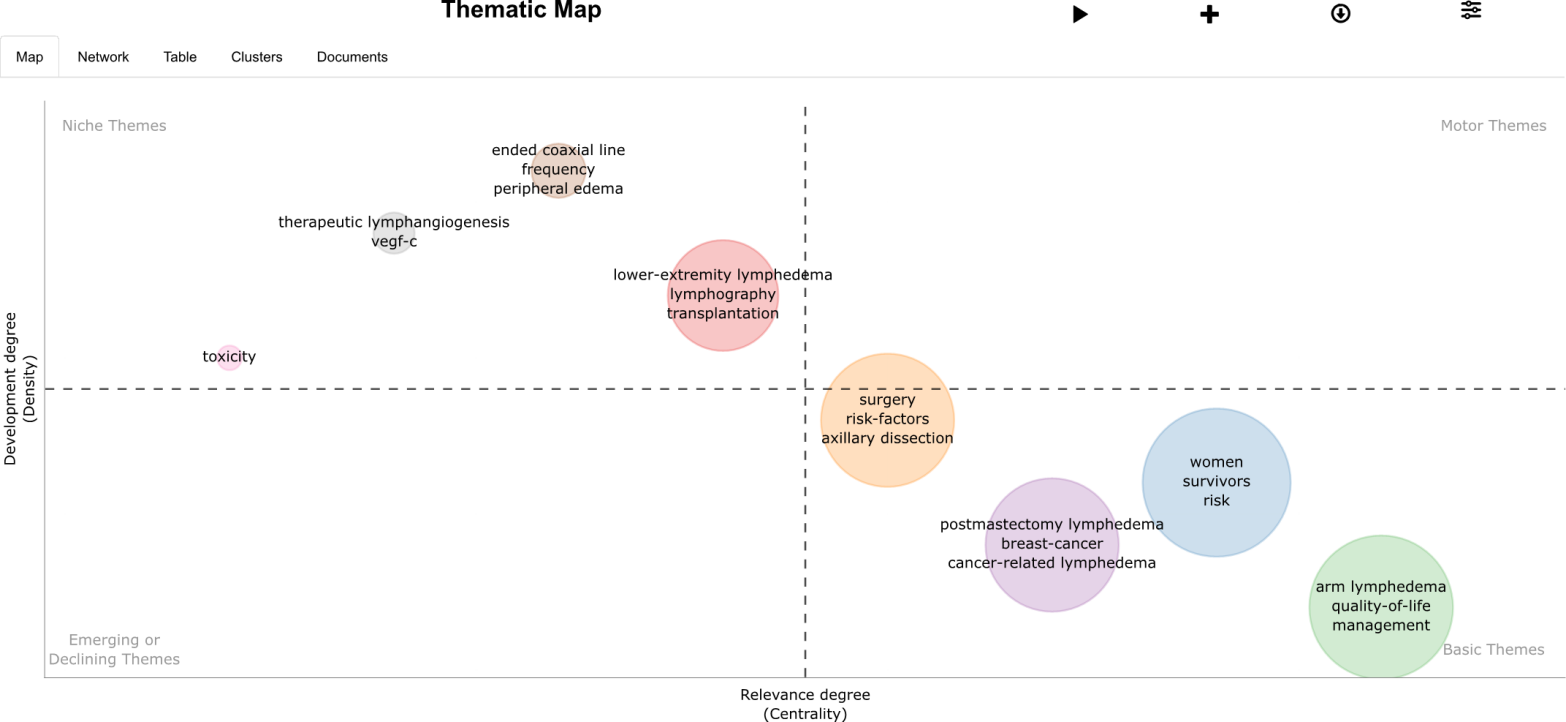
The thematic map of research areas.

The thematic map of BCRL research (**Figure 12**) provides a comprehensive overview. The bottom-right corner represents foundational themes, such as surgery, women, postmastectomy lymphedema, and arm lymphedema, forming the core of this research field. The top-left quadrant highlights niche themes, such as therapeutic lymphangiogenesis and lower extremity lymphedema, reflecting specialized or cutting-edge research directions. This analysis identifies dominant trends and emerging research priorities, offering valuable perspectives for understanding the current state of BCRL research and guiding future studies.

### 3.8 Analysis of research categories

Eighty-one research categories were identified in this field. The top five categories by publication volume are listed in **Table 9**: “Oncology” (967 publications), “Cell Biology” (308 publications), “Biochemistry and Molecular Biology” (301 publications), “Endocrinology and Metabolism” (286 publications), and “Pathology” (170 publications).

**Table 9:** The top 10 active research categories.

Research areas	Record count	Percentage
Oncology	364	39.608
Surgery	208	22.633
Physiology	159	17.301
Research Experimental Medicine	130	14.146
Rehabilitation	100	10.881
Health Care Sciences Services	62	6.746
General Internal Medicine	52	5.658
Nursing	42	4.570
Obstetrics Gynecology	42	4.570
Immunology	40	4.353

## 4 Discussion

Unlike traditional literature reviews, bibliometric analysis systematically examines literature within a specific field (29), offering a structured approach to uncover collaboration networks, research trends, key interest areas, and potential developments. In this comprehensive bibliometric study, conducted from January 1, 2000, to November 30, 2023, we utilized R Studio and VOSviewer to map these elements in the context of BCRL. CiteSpace was employed to identify the top 25 citations and keywords with the strongest citation bursts. The analysis covered 919 records published in 255 journals by 3,550 authors affiliated with 1,163 institutions across 52 countries/regions. The findings reveal an increasing trend in publications, peaking in 2022 with 99 outputs, reflecting growing academic interest in the field. While the 2023 output appears lower, this is attributed to the cutoff date of November 30, 2023. The significant global impact of breast cancer, with 2.26 million new cases reported in 2020 (20), underscores the importance of research into its complications, such as lymphedema. As the most diagnosed cancer globally, breast cancer presents an urgent need for studies aimed at preventing and managing lymphedema to improve patient outcomes and quality of life. The increasing scholarly output mirrors deeper investigations into breast cancer survivors’ prognosis and a more nuanced understanding of lymphedema as a treatment-related complication.

The USA leads in publications, citations, and centrality, underscoring its influence and extensive international collaborations. Notable contributions from developing countries, especially China, signal promising progress in the field. However, disparities in publication volume between the USA and other countries/regions remain evident (30). Asian research teams should enhance their global impact by strengthening collaborations with European and American counterparts. Furthermore, institutions active in this field should foster stronger interconnections to support intensive studies on BCRL. Journals in this domain primarily focus on surgical procedures and cancer care, with “Lymphology” prominently emphasizing immunological aspects. Leading authors, such as Kathryn H. Schmitz and Alphonse G. Taghian, have made significant contributions, particularly in assessing the safety of upper-body exercise for breast cancer survivors with lymphedema and exploring the role of physical activity in prevention and rehabilitation (31, 32). The most cited articles in this bibliometric analysis focus on key aspects of BCRL, including arm edema in breast cancer patients, weightlifting safety, lymphedema management, and quality of life. These foundational works, primarily literature reviews published before 2012, continue to influence current research directions. Their enduring relevance highlights their importance as cornerstones in understanding and addressing BCRL, providing a basis for future advancements in the field.

Keyword analysis from 2000 to 2023 identified research hotspots and projected trends, particularly in postoperative arm swelling, the relationship between lymphedema and quality of life, and the effectiveness of management strategies. BCRL arises from treatments like surgery and radiotherapy as well as metastases, necessitating comprehensive management encompassing prevention, diagnosis, and treatment (33). Prevention begins with postoperative screening for BCRL risks, such as surgical techniques for lymph node resection, radiation exposure, and obesity (34). Education enables patients to recognize early symptoms (35), such as limb swelling and restricted mobility. Preventive measures include maintaining skin hygiene, avoiding trauma, and using compression cannulas to delay the onset of arm edema within the first year post-surgery (36, 37).

Diagnosis relies on self-reported symptoms, clinical evaluations, and the International Society of Lymphology (ISL) staging system (38), which classifies lymphedema into stages 0–III. Technological advancements, such as bioimpedance spectroscopy and low-level laser therapy, have improved early detection and management. Treatment focuses on early intervention, emphasizing gradual rehabilitation and avoiding strenuous activities (39). Complex decongestive therapy (CDT)—comprising manual lymph drainage, compression garments, skincare, and exercise—remains the primary treatment (40). However, evidence suggests no definitive superiority of any single treatment in reducing BCRL volume, indicating the need for further research to understand the efficacy of these components across lymphedema stages (41). Rehabilitation strategies, including deep breathing, aerobic exercises, and psychological support, play critical roles in improving patient outcomes and quality of life (42).

This study has several limitations. The limited time frame for data extraction excluded the most recent publications, and the focus on English-language journals may have missed valuable insights from non-English research. Using only the Web of Science (WOS) database may have overlooked relevant works in discipline-specific journals. Manual searches for author affiliation data introduced potential bias, and reliance on VOSviewer for first-author cocitation may have limited the scope of the analysis. Additionally, older articles may not fully represent the current research landscape.

Future studies should address these limitations by including non-English sources and additional databases, while adopting interdisciplinary approaches (43). Research should focus on prevention strategies, improving diagnostic methods, and evaluating long-term treatment efficacy. Exploring the impact of emerging technologies, such as telemedicine and wearable devices, as well as leveraging big data and AI (44), could improve BCRL management and patient quality of life. Collaboration across oncology, rehabilitation, and psychology fields is essential to address the multifaceted nature of BCRL.

## 5 Conclusion

This bibliometric analysis of breast cancer-related lymphedema research from 2000 to 2023 highlights a significant rise in scholarly activity over the past two decades. The USA leads in publication volume and influence, with *Lymphology, Breast Cancer Research and Treatment, and Plastic and Reconstructive Surgery* as the most productive journals. Alphonse G. Taghian stands out as a key contributor in this field. Despite advancements, challenges remain in developing effective treatments and prevention strategies. With the growing prevalence of breast cancer, addressing BCRL through targeted research in risk screening, prevention, and management is increasingly critical. Collaborative efforts will be essential to improving outcomes for affected patients.

## Data Availability

The original contributions presented in the study are included in the article/supplementary material. Further inquiries can be directed to the corresponding authors.
